# Injury of the Deep Branch of the Ulnar Nerve: A Sonographic Diagnosis

**DOI:** 10.5334/jbsr.3103

**Published:** 2023-04-28

**Authors:** Lorthioir Félicie, Clermont Didier, Gudelj Maxime

**Affiliations:** 1 CHU Liège, BE; 2 Citadelle L’Hopital, BE

**Keywords:** ultrasonography, ulnar-palmar wrist region, deep branch of the ulnar nerve

## Abstract

**Teaching Point:** The ulnar-palmar wrist region has a unique anatomy with some specific pathologies, easily assessed with high-frequency ultrasound.

## Case History

A 21-year-old man was admitted in our emergency department with a deep wound (knife injury) on the right-hand palmar region. That injury was sutured and no imaging exam was performed. One month later the patient complained of an impairment of the fourth finger adduction and flexion with first commissure amyotrophy. A lesion on the deep branch of the ulnar nerve (DBUN) was suspected by the orthopedic surgeon. An elective impairment of DBUN was diagnosed on electromyography (EMG).

**Figure 1:**
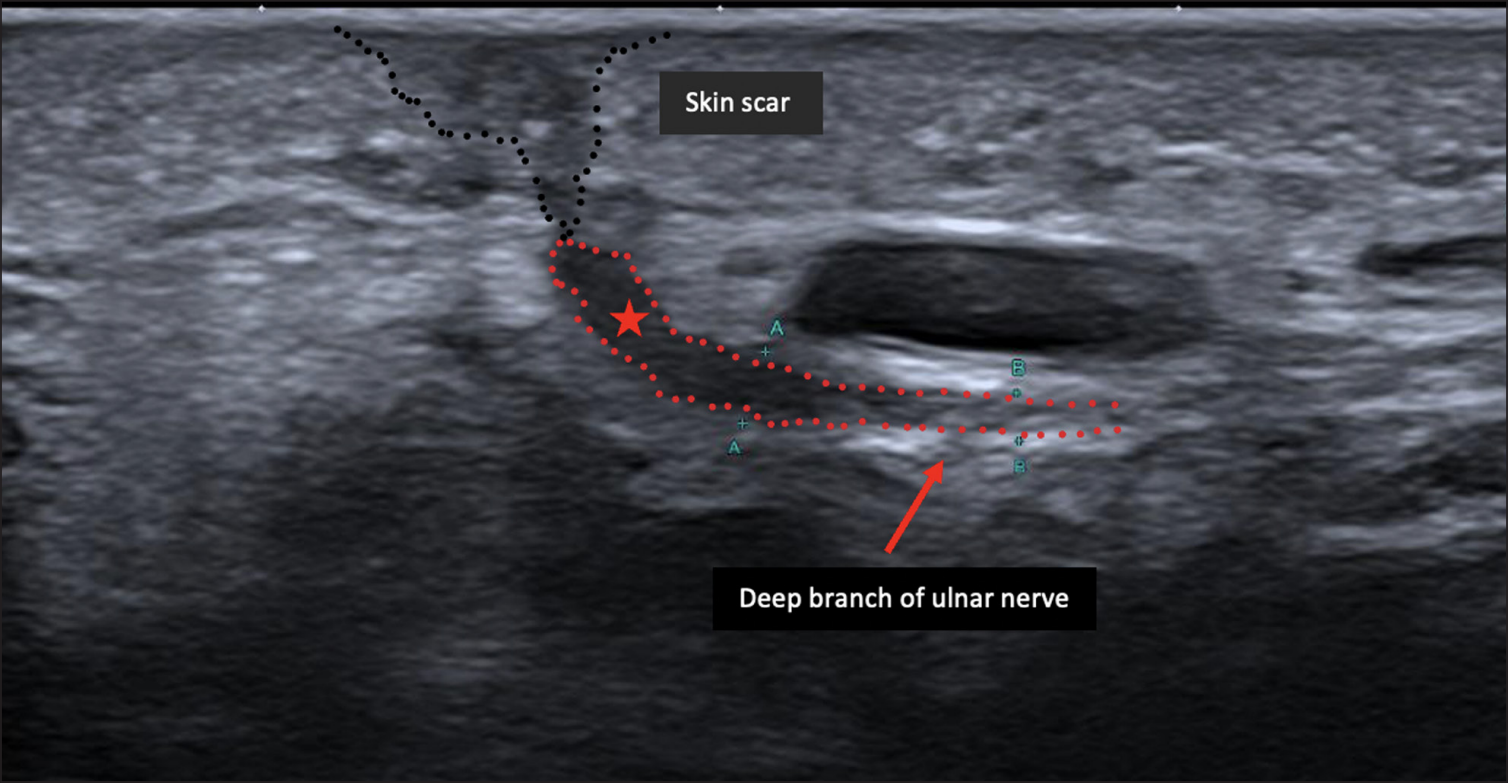


**Figure 2:**
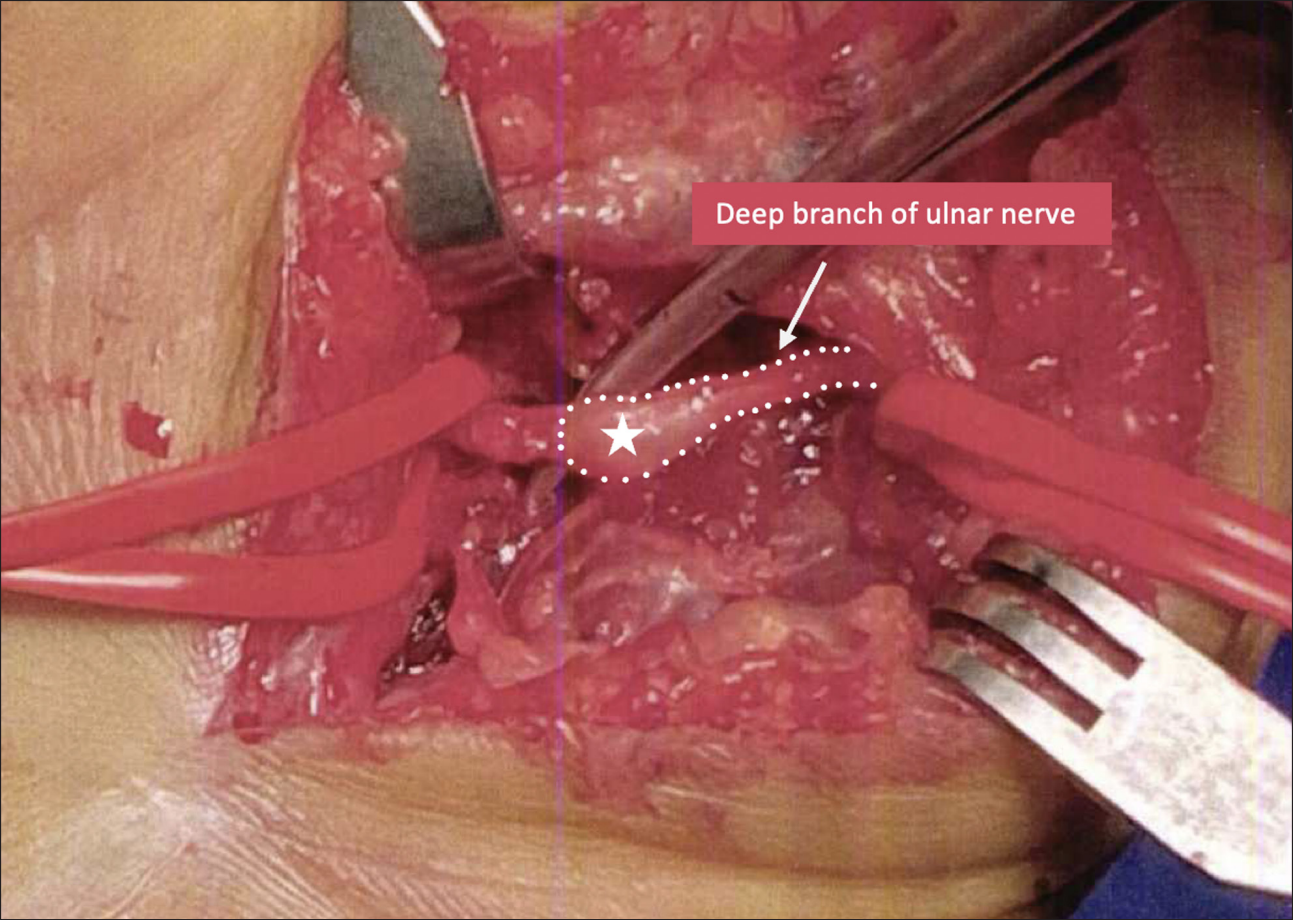


An ultrasound (US) was performed and showed DBUN (arrow) transection underneath the scar (black dotted line) with a hypo-echoic neuroma (star) at the proximal end (Figure 1) without any hyperemia on Power Doppler. The patient underwent a neuroma surgical resection and nerve suture (Figure 2) with a progressive improvement of symptomatology over a few months.

## Comments

Injury of the ulnar nerve and its branches is not uncommon. Due to its superficial location, it is prone to penetrating and iatrogenic injuries. Prognosis depends on early diagnosis and effective treatment.

Clinical examination and EMG findings are able to assess nerve involvement and can determine the most likely location of the injury but cannot provide any anatomical information.

US performed with high-resolution broadband linear transducer and magnetic resonance imaging (MRI) are the Gold Standard imaging methods for superficial nerve injuries diagnosis.

Commonly encountered pathologies include compressive, tumoral, and traumatic injuries. Traumatic injuries appear as partial or full nerve transection with or without development of a hypoechoic ovoid neuroma.

Unfortunately, radiologists underestimate the opportunity of US in the assessment of the ulnar- palmar wrist region (UPWR). Indeed, due to its accessibility, low cost, absence of contraindications, opportunity of dynamic and comparative study, and excellent spatial resolution, US is able to provide a careful and accurate evaluation of the UPWR and ulnar nerve and its branches [1].

Traumatic nerve injury diagnosis can be challenging in acute setting. EMG cannot be use in the first two weeks because Wallerian degeneration must be awaited to observe the denervation due to axonal damage. Peripheral nerve imaging with ultrasound, in the acute phase, can directly showing nerve transection and fill this ‘electrodiagnostic time gap.’ Nerve ultrasound is recommended, in acute phase of peripheral nerve trauma, particularly when there are clear clinical proven of nerve injury.
